# Evodiamine Stabilizes Topoisomerase I-DNA Cleavable Complex to Inhibit Topoisomerase I Activity

**DOI:** 10.3390/molecules14041342

**Published:** 2009-03-27

**Authors:** Agnes L.-F. Chan, Wen-Shin Chang, Li-Min Chen, Chi-Ming Lee, Chiao-En Chen, Chun-Mao Lin, Jau-Lang Hwang

**Affiliations:** 1 Pharmacy Department, Chi Mei Medical Center, Tainan 710, Taiwan; E-mail: cmh5500@mail.chimei.org.tw (A-F.C.); 2 College of Pharmacy, Taipei Medical University, Taipei 110, Taiwan; E-mail: b507094042@tmu.edu.tw (W-S.C.); 3 College of Medicine, Taipei Medical University, Taipei 110, Taiwan; E-mails: b104094024@tmu.edu.tw (L-M.C.), januslee@tmu.edu.tw (C-M.L.); 4 Institute of Molecular Biology, Academia Sinica, Nangkang, Taipei 115, Taiwan; E-mails: joan1118@imb.sinica.edu.tw (C-N.C.), jh@ccvax.sinica.edu.tw (J.H.); 5 Orthopedics Research Center, Taipei Medical University Hospital, Taipei 110, Taiwan

**Keywords:** Evodiamine, Topoisomerase, Covalent complex.

## Abstract

Evodiamine (EVO), an alkaloidal compound isolated from *Evodia rutaecarpa* (Juss*.*), has been reported to affect many physiological functions. Topoisomerase inhibitors have been developed in a variety of clinical applications. In the present study, we report the topoisomerase I (TopI) inhibitory activity of EVO, which may have properties that lead to improved therapeutic benefits. EVO is able to inhibit supercoiled plasmid DNA relaxation catalyzed by TopI. Upon treatment 0~10 μM EVO TopI was depleted in MCF-7 breast cancer cells in a concentration-dependent and time-dependent manner in 0~120 min. A K-SDS precipitation assay was performed to measure the extent of Top I-trapped chromosomal DNA. The ability of EVO to cause the formation of a TopI-DNA complex increased in a concentration-dependent manner, in that the DNA trapped increased by 24.2% in cells treated with 30 μM. The results suggest that EVO inhibits TopI by stabilizing the enzyme and DNA covalent complex.

## 1. Introduction

DNA topoisomerase enzymes regulate the topological state of DNA that is crucial for initiation and elongation during DNA synthesis. Topoisomerase I (TopI) produces a single strand break in DNA, allowing relaxation of DNA during its replication. The single strand break is then religated, thus restoring the DNA double strands. The enzymatic mechanism involves two sequential transesterification reactions [1]. In the cleavage reaction, the active site tyrosine (Tyr723 in human TopI) acts as a nucleophile. A phenolic oxygen attacks a DNA phosphodiester bond, forming an intermediate in which the 3′ end of the broken strand is covalently attached by an O^4^-phosphodiester bond to TopI tyrosine. The religation step consists of transesterification involving a nucleophilic attack by the hydroxyl oxygen at the 5′ end of the broken strand. The equilibrium constant of the breakage and closure reactions is close to unity, and the reaction is reversible.

Some TopI- and TopII-targeted drugs are reported to stabilize the covalent topoisomerase-DNA complex, thereby preventing religation [2]. The TopI reaction intermediate consists of the enzyme covalently linked to a nicked DNA molecule, known as a “cleavable complex”. Covalently bound TopI-DNA complexes can be trapped and purified because the enzymatic religation is no longer functional. Camptothecin (CPT) is a representative drug that targets DNA TopI through trapping a covalent intermediate between TopI and DNA.

The inhibitor-trapped TopI-cleavable complex triggers replication fork arrest and breakage to generate a DNA break that is responsible for the G2/M arrest and activation of DNA damage signals including nuclear factor κB activation, p53 upregulation, Chk1 phosphorylation, and ATM/ATR activation [3]. The phosphorylated form of DNA-PK increased after treatment with a topoisomerase inhibitor [4]. Topoisomerase inhibitors have been developed for antitumor [5, 6], antiviral [7], antibacterial [8], anti-epileptic [9], and immunomodulation [10] applications.

Evodiamine (EVO), an alkaloidal compound isolated from *Evodia rutaecarpa* (Juss.), has been reported to possess many physiological functions including vasorelaxation, antiobesity [11], anticancer [12], and anti-inflammatory [13] effects. Evidence showed that EVO induced apoptosis through an accumulation of the cell cycle at the G2/M phase and initiation of apoptosis [14]. The EVO derivatives rutaecarpine, evodiamide, 14-formyldihydrorutaecarpine and dehydroevodiamine, are usually found as contents in preparations [15]. In this study, we report the TopI-DNA trapping activity by EVO, which suggests beneficial effects of EVO for the development of a variety of therapeutic applications.

## 2. Results and Discussion

### 2.1. Growth inhibition of EVO

**Figure 1:**
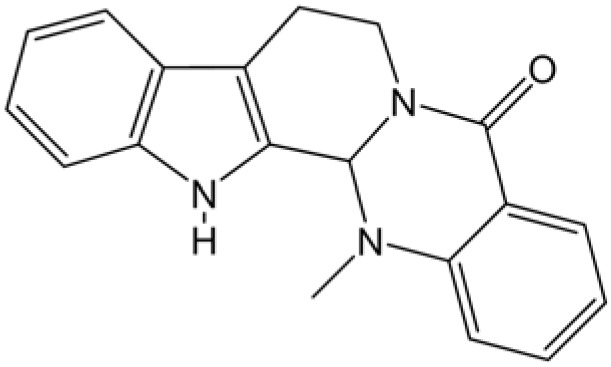
Chemical structure of evodiamine.

The cytotoxic abilities of EVO (Figure 1) and CPT were tested against human breast MCF-7 carcinoma cells, which express high levels of TopI. CPT was used as a reference drug. Both compounds displayed efficient cytotoxicity after 24 h of drug exposure in the MTT assay. The IC_50_ values were 3.23 μM for CPT and 6.02 μM for EVO. EVO was slightly less cytotoxic than CPT against breast MCF-7 carcinoma cells.

### 2.2. Inhibition of supercoiled DNA relaxation from Top I catalysis by EVO

**Figure 2:**
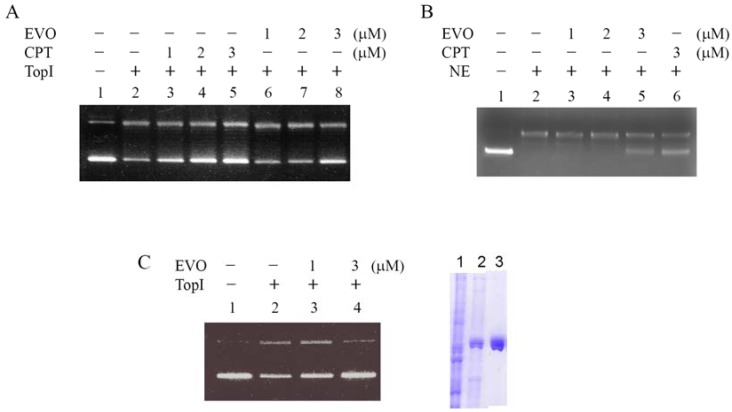
Inhibitory activity of evodiamine (EVO) on topoisomerase I (TopI). (A) Both camptothecin (CPT) and EVO (1~3 μM) protected DNA from Vaccinia Top I conversion of supercoiled DNA to relaxed closed circular DNA. pCDNA3 (0.2 μg) plasmid DNA was incubated at 37 °C for 30 min with TopI (0.5 units) from the Vaccinia virus in the presence or absence of 1~3 μM inhibitors. (B) The reactions with same conditions as adopted in (A) except using nuclear extract (NE) of MCF-7 cells as enzyme source. (C) The same assay conditions as adopted in (A) except using purified recombinant human TopI as enzyme source (left). Recombinant human TopI was obtained using the baculovirus expression system (right, lane 1: cell lysate, lane 2: partial purified fraction, and lane 3: Ni-NTA column purified protein).

TopI is known to relax the supercoiled plasmid DNA to an open circular form *in vitro* and *in vivo*. CPT and EVO inhibition of supercoiled DNA relaxation *in vitro* was evaluated. Vaccinia TopI's induction of supercoiled pCDNA3 plasmid relaxation was employed as the assay system, and the results are shown in Figure 2. Supercoiled DNA migrated faster on the agarose gel than the relaxed circular DNA, as shown in the control (Figure 2, lanes 1 and 2). CPT treatments retained a greater amount of uncatalytic supercoiled DNA in a concentration-dependent manner (lanes 3~5, 1~3 μM). EVO also displayed inhibitory activity on TopI catalytic relaxation in a concentration-dependent manner (lanes 6~8, 1~3 μM). These results suggest that EVO is able to inhibit supercoiled plasmid DNA relaxation catalyzed by TopI. Using nuclear extract of MCF-7 cells as enzyme source, EVO also showed inhibitory activity on DNA relaxation in a concentration-dependent manner (Figure 2B, lanes 3~5, 1~3 μM). To further confirm the inhibitory activity on human topoisomerase I (hTopI), recombinant hTopI protein was expressed and purified using the baculovirus expression system (Figure 2C, right panel) for the relaxation assays. Again, EVO revealed inhibitory activity on DNA relaxation catalyzed by hTopI (Figure 2C, lanes 3~4).

The data reveal that evodiamine is less effective in inhibiting vaccinia TopI than hTopI. Even Vaccinia virus DNA TopI has been reported to be resistant to the effects of camptothecin [16]; it has also been proposed a potential interaction site for camptothecin [17]. With a reduced amount of enzyme in the assay mixture we demonstrated a detectable effect of camptothecin in inhibiting Vaccinia TopI DNA relaxation.

### 2.3. EVO depletes the Top I protein

**Figure 3:**
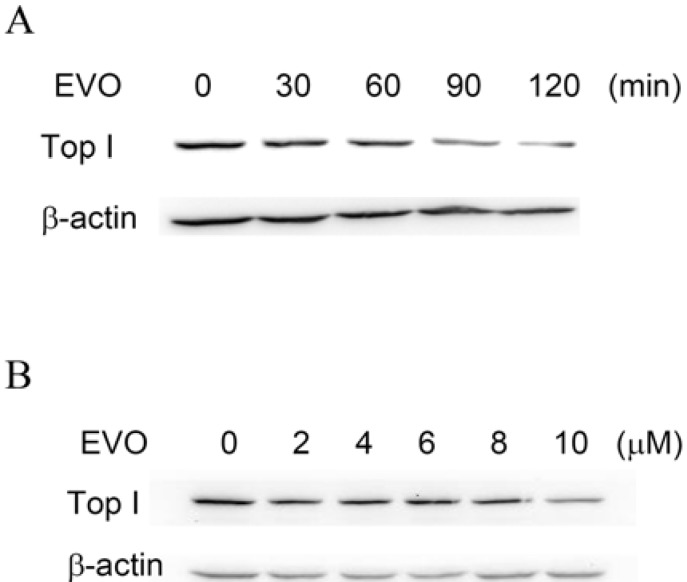
Topoisomerase I (TopI) depletion by evodiamine (EVO) in MCF-7 cells. (A) MCF-7 cells were treated with EVO (10 μM) for 0~120 min. (B) MCF-7 cells were treated with various concentrations of EVO (0~10 μM) for 60 min. Protein levels of TopI were examined by immunoblotting. β-Actin with constant expression was used as the internal control.

A depletion assay was performed to confirm the effect of EVO on DNA topological catalysis of TopI. This assay was based on the ability of CPT to trap TopI and DNA to form a CPT-TopI-DNA triple complex, to see if EVO inhibits TopI activity through a similar mechanism. The degree to which depletion occurs depends on the extent to which TopI binds DNA, which is a function of EVO sensitivity. MCF-7 cells maintained in normal conditions expressed a detectable level of TopI protein. MCF-7 cells were treated with EVO for 0~120 min. The protein levels of TopI were examined by immunoblotting. The levels of TopI in EVO treatments (10 μM) were depleted in a time-dependent manner, in that the protein level decreased to < 20% with 120-min treatment in comparison to the untreated control (Figure 3A). Depletion of TopI protein by EVO was also detected in a concentration-dependent manner after 1 h of treatment. The relative level of DNA-unlinked TopI protein after treatment with 0~10 μM EVO decreased to < 40% versus the control (Figure 3B). β-Actin with constant expression was used as the internal control.

### 2.4. TopI-DNA complex-trapping activity in MCF-7 cells

**Figure 4:**
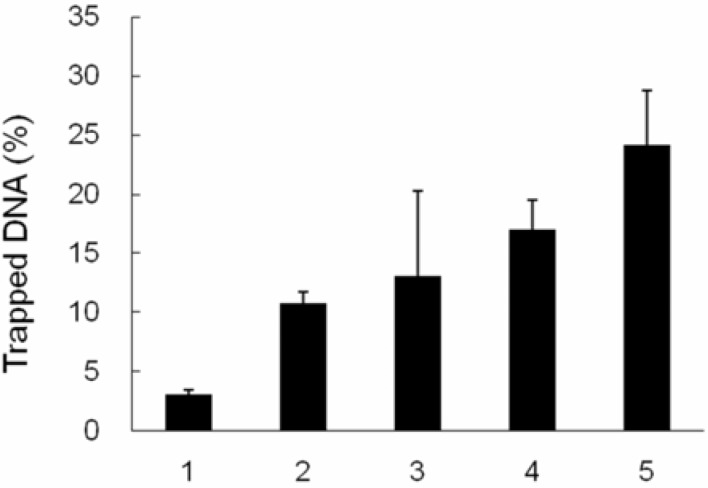
Topoisomerase I (TopI)-DNA complex trapping activity of evodiamine (EVO). 3H-Thymidine-labeled MCF-7 cells were treated with various concentrations (0~30 μM) of EVO for 60 min. K-SDS precipitation was performed to trap the TopI-DNA complex. Radioactivity counts of trapped DNA were determined using a scintillation counter.

TopI can be trapped in a covalent complex with DNA by adding protein denaturants. Capturing the covalent intermediate or cleavable complex is difficult because the intermediate has a relatively short lifetime. KCl/SDS can precipitate the protein, but not DNA, except when it is linked to a protein. Therefore, the amount of precipitated DNA reflects the EVO-trapping activity. ^3^H-thymidine-labeled cells were treated with EVO (0, 5, 10, 20, and 30 μM, respectively) for 60 min, and *in vivo* K-SDS precipitation was performed. Only 3% of the ^3^H-thymidine-labeled DNA was trapped in a covalent complex with TopI in 0 μM-treated MCF-7 cells. The DNA trapped by EVO increased to 24.2% with 30 μM treatment (Figure 4). This result indicates that the ability of EVO to cause the formation of the TopI-DNA complex increased with EVO treatment in a dose-dependent manner.

### 2.5. Proposed TopI-inhibition mechanism of EVO

**Figure 5:**
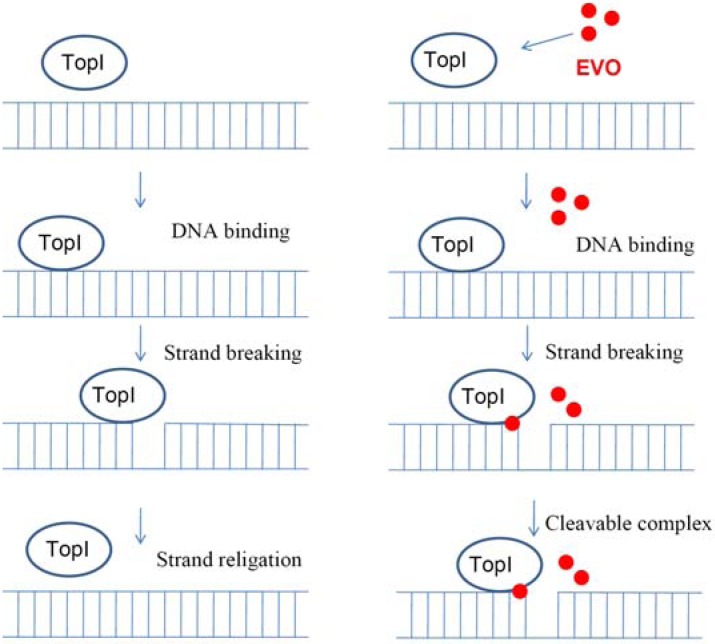
Proposed mechanism of evodiamine (EVO) inhibition of topoisomerase I (TopI) by stabilizing a covalent complex.

We propose the mechanism of EVO as a TopI inhibitor. TopI catalyzes DNA strand breakage and religation during the procedure of replication and transcription (Figure 5, left panel). The effect of EVO acts by stabilizing the covalent complex between TopI and DNA (Figure 5, right panel), which results in free-form TopI depletion and a barrier to DNA replication and transcription

There are several problems with CPT-derived anticancer agents despite their clinical success. Multidrug resistance (MDR) results from their intracellular concentration being greatly reduced by efflux pumps in a wide variety of tissues which is conferred by P-glycoprotein overexpression [18]. 

Additionally, all CPTs are substrates for the pump known as breast cancer-resistant protein [19]. EVO isolated from the fruits of *Evodia officinalis* by Xu *et al*. did not reveal inhibitory effect on DNA TopI in their assay system [20]. The action of EVO as a TopI inhibitor for anticancer chemotherapy is expected to be greatly enhanced, according to the results of the present study. It may have properties that lead to improved therapeutic benefits to patients with CPT tolerance because EVO has shown anticancer effects in adriamycin-resistant human breast cancer cells [21]. The targeting site of EVO efficacy in the covalent complex remains poorly understood, but studies of the interaction of CPT derivatives have revealed clues to the process, not only due to their structural similarities, but also to the comparable TopI-DNA complex trapping activities.

## 3. Experimental

### 3.1 Materials

Recombinant human DNA TopI was obtained using the baculovirus expression system as described [22]. DNA TopI from the vaccinia virus was purchased from Epicentre Biotechnologies (Madison, WI, USA). Evodiamine (purity: >99%) and camptothecin (purity: ~95%) were purchased from Sigma (St. Louis, MO, USA). 

### 3.2 Cell culture

MCF-7 breast cancer cells (ATCC HTB-22) were cultured in Dulbecco's modified Eagle medium (DMEM) supplemented with 10% heat-inactivated fetal bovine serum (FBS), and 100 μg/mL penicillin-streptomycin. Conditions were maintained at 37 °C in a humidified 95% air/5% CO_2_ incubator.

### 3.3. Preparation of Nuclear Extract

Human MCF-7 cells (1 × 10^7^) were resuspended in lysis buffer (400 μL, 10 mmol/L HEPES [*N*-2-hydroxyethylpiperazine-*N*-2-ethanesulfonic acid], 10 mmol/L KCl, 1.5 mmol/L MgCl_2_, and 0.5 mmol/L dithiothreitol [DTT], pH 7.9) with 0.2% Nonidet P-40 (NP-40) and protease inhibitor cocktail for 1 min on ice. After being microcentrifuged for 1 min at 2,500×*g*, the supernatants were collected as cytoplasmic protein extracts. The pellets were washed with lysis buffer without NP-40, resuspended in extraction buffer (150 μL, 20 mmol/L HEPES, pH 7.9, 420 mmol/L NaCl, 0.5 mmol/L DTT, 0.2 mmol/L EDTA, and 25% glycerol), and incubated for 20 min on ice. After at 12,000×*g* for 10 min, the supernatants were collected as nuclear protein extracts, and aliquots were stored at −70 °C until use in relaxation assays.

### 3.4. Recombinant human TopI (hTopI) protein expression and purification

cDNAs encoding full-length hTopI were subcloned into the baculoviral expression vector pFastBac HTa and pFastBac HTc. The bacmid constructs were prepared using the Bac-to-Bac baculovirus expression system protocol (Invitrogen). To express and purify the recombinant hTopI, recombinant baculoviral stock were used to infect 2 x 10^7^ Sf21 insect cells per 140-mm plate. Infected cells were cultured at 27 °C for 3 days. The Ni-NTA column/imidazole was used for the hTopI fractionation. Aliquots were stored at −70 °C until use in relaxation assays [23]. 

### 3.5. TopI-catalyzed supercoiled DNA relaxation

The inhibitory effect of CPT and EVO on supercoiled DNA strand breakage caused by TopI was evaluated. pCDNA3 plasmid DNA (200 ng) was incubated at 37 °C for 30 min in a reaction solution (50 mM Tris-acetate, 100 mM NaCl, 2.5 mM MgCl_2_, and 0.1 mM EDTA; pH 7.5) in the presence or absence of 0~3.0 μM inhibitor in a final volume of 20 μL. The conversion of the covalently closed circular double-stranded supercoiled DNA to a relaxed form was used to evaluate DNA strand breakage induced by TopI. Samples were loaded onto a 1% agarose gel, and electrophoresis was performed in TAE buffer (40 mM Tris-acetate and 1 mM EDTA), then the gel was photographed under transmitted ultraviolet light [24, 25]. 

### 3.6. KCl/SDS precipitation assay of the covalent TopI-DNA complex

The formation of a cleavable complex in intact cells was quantified by a K-sodium dodecylsulfate (SDS) precipitation technique, a modified procedure described previously by Yoshinari *et al.* [26]. Cellular DNA was labeled by adding ^3^H-thymidine to the medium to a final concentration of 10 μCi/mL. After an overnight incubation, cells were plated to a density of 1 × 10^5^ cells/well in a 24-well plate for another overnight incubation, and treated with various concentrations of EVO (0~30 μM) for 60 min. The medium was removed from each well, and cells were washed with phosphate-buffered saline (PBS) and lysed with 1 mL of prewarmed (65 °C) lysis solution (1.25% SDS, 5 mM EDTA, 0.4 mg/mL salmon sperm DNA). Lysate was sheared using a 21-gauge needle. The samples as background control were treated by the above procedure but with proteinase K (400 μg/mL) in the lysis buffer, and were incubated at 50 °C for 2 h. KCl (325 mM), at 250 μl, was added to each sample, vortexed vigorously, cooled on ice for 10 min, and centrifuged at 2,500 rpm for 10 min at 4 °C. The pellet was washed twice in wash solution (1 mL, 10 mM Tris-HCl, 100 mM KCl, 1 mM EDTA, and 0.1 mg/mL salmon sperm DNA) and incubated at 65 °C for 10 min, cooled on ice, then centrifuged at 2,500 rpm for 10 min. The pellet was resuspended in prewarmed H_2_O (400 μL, 65 °C), combined with scintillation liquid (4 mL) and the radioactivity counts were determined.

### 3.7. Detection of the protein levels of TopI

MCF-7 cells grown in monolayer culture that were 50%~80% confluent were scraped and collected by centrifugation. The cell pellets were lysed in 10 volumes of a 2x SDS sample buffer (20% glycerol, 10% β-mercaptoethanol, 6% SDS, and 125 mM Tris; pH 6.8) and heated in a boiling water bath for 5 min. Samples of each lysate (50 μg proteins/lane) were separated electrophoretically in a 7.5% SDS polyacrylamide gel and electro-transferred to a polyvinylidene difluoride (PVDF) membrane (Immobilon^P^, Millipore, Bedford, MA, USA). 

The membrane was incubated with the primary antibody (rabbit anti-human topoisomerase I polyclonal antisera) [27] at room temperature for 2 h, and then incubated with a horseradish peroxidase-conjugated secondary immunoglobulin G (IgG) antibody; the immunoreactive bands were visualized with enhanced chemiluminescent reagents (Amersham, Buckinghamshire, UK), and photographed using the gel documentation system (UVP, Upland, CA).

### 3.8. MTT cell viability assay

The MTT [3-(4,5-dimethylthiazol-2-yl)-2,5-diphenyltetrazolium bromide] assay to test the cytotoxicity of reagents and cell viability was described previously [28]. MCF-7 cells (5000 cells/well) were grown on a 96-well plate supplemented with DMEM medium (with 1% FBS) for 24 h. Cells were treated with CPT or EVO (0~30 μM), and the viability was determined by the reduction of MTT. An MTT stock solution (5 mg of MTT/mL of PBS) was added to the growing cultures (at a final concentration of 0.5 mg/mL). The OD was measured with a spectrophotometer (Thermo Varioskan Flash, Vantaa, Finland) at 560 nm. A blank with DMSO alone was measured and subtracted from all values.

## 4. Conclusions

EVO is reported to have various pharmacological effects, but this is the first demonstration of EVO as a TopI inhibitor. EVO acts by stabilizing the covalent complex between TopI and DNA during the procedure of TopI-catalyzed DNA strand breakage and religation, which results in a barrier to DNA replication and transcription.
